# Correction for Self-Selection in Breast Cancer Screening. Comment on Dibden et al. Worldwide Review and Meta-Analysis of Cohort Studies Measuring the Effect of Mammography Screening Programmes on Incidence-Based Breast Cancer Mortality. *Cancers* 2020, *12*, 976

**DOI:** 10.3390/cancers15184576

**Published:** 2023-09-15

**Authors:** Martin J. Yaffe

**Affiliations:** 1 Physical Sciences Platform, Sunnybrook Research Institute, Toronto, ON M4N 3M5, Canada; martin.yaffe@sri.utoronto.ca; Tel.: +1-416-480-5715; 2 Imaging Research Program, Ontario Institute for Cancer Research, Toronto, ON M5G 0A3, Canada

**Keywords:** breast cancer, screening, observational studies, self-selection bias, correction

## Abstract

**Simple Summary:**

Studies looking at the reduction of breast cancer deaths from screenings that are not randomized may suffer bias because of “self selection”. This occurs when even before screening takes place, people who choose to be screened are already at lower risk of dying than those who do not participate. A publication by Dibden et al. applied a correction to remove the effect of self-selection. There is concern, though, that because the correction was developed using data from a group of women in Sweden, where the healthcare system and people’s attitudes and behavior may not be the same, it may not be suitable when applied to studies on women in other countries such as Canada. In fact, the authors of the Canadian study checked for and found no evidence of self-selection bias.

**Abstract:**

Observational studies of cancer screening are subject to bias associated with the self-selection of screening participants for whom the underlying probability of cancer death may be different from those who do not participate. Dibden et al. reviewed data on mortality reduction from 27 observational studies of mammography screening expressed in terms of relative risk for women who were screened versus not screened. Results were given, both unadjusted and after application of a correction for self-selection. The correction was based on a constant (1.17)—the ratio of risks of death in screening non-attenders versus those not invited, derived from a Swedish study. For some of the studies this correction had a large effect in diminishing the measured mortality benefit associated with screening. In particular, application to The Pan-Canadian Study of Mammography Screening, a study whose authors had previously tested for and found no evidence of self-selection bias, caused the estimated benefit to decrease from 40% to 10%. The appropriateness of applying a correction based on a constant to a population whose healthcare environment and screening participation rates are very different from those from which it was derived is questionable.

## 1. Introduction

In the review by Dibden et al. (Worldwide Review and Meta-Analysis of Cohort Studies Measuring the Effect of Mammography Screening Programmes on Incidence-Based Breast Cancer Mortality), published in 2020 in this journal, the authors evaluated data from 27 different observational studies of mammography screening using breast cancer-specific mortality as an endpoint [1]. They applied a correction for self-selection bias, based on previously published evidence that mortality from individuals invited to be screened, but who did not attend screening, is often higher than that for uninvited individuals. The correction, originally developed by Duffy et al. [2], utilizes a value referred to as D_r_ and described by Dibden et al. as “the risk of death in non-attenders versus uninvited from an appropriate external source”. A constant value of 1.17 for D_r_ was applied in the correction for all studies, the value taken from a study by the Swedish Organised Service Screening Evaluation Group (SOSSEG) [3]. 

The authors applied a correction based on this constant to the unadjusted relative risks for breast cancer mortality in women attending screening compared to non-attendees in the Pan-Canadian Study of Mammography Screening: a large (20.1 million person-y) observational study conducted across multiple provincial screening programs in Canada [4]. The correction caused the initial RR of 0.6 (40% reduced mortality) for women 40–79 to be increased to 0.90 (a 50% increase in RR). The across-the-board application of a constant value of D_r_ determined in another environment raises some concerns.

## 2. Discussion

In the correction used by Dibden et al., D_r_ is the ratio, as determined from a reference population (in this case, SOSSEG), of the risk of death from breast cancer in non-attenders (numerator) to the risk in women not invited for screening (denominator). Presumably, in this Swedish reference population, the uninvited group comprised a mixture of those who would have attended if they had been invited to be screened and those who would not have accepted the invitation had it been given and the denominator would be the weighted sum of the risks associated with these two components. If these two subgroups had different risks of dying of breast cancer, the denominator and, therefore, the value of D_r_, would depend on the proportion of Swedish women who would have attended if invited (participation rate). This proportion and also possibly the inherent risks of dying of breast cancer as an attendee or non-attendee of screening are likely to depend highly on the screening environment and the health care system. Certainly, participation rates were quite different between the Canadian and Swedish cohorts; the authors described the participation rate in Canada as 44%, while that in Sweden was 80% [1]. Factors associated with access to screening, efficacy of the intervention and attitudes toward public health interventions could vary greatly from one country to another or even between regions. Similarly, socioeconomic differences could affect behavior and outcomes. For this reason, it is unlikely that a value of D_r_ derived from a Swedish population would appropriately apply to women living in another environment.

The authors note the heterogeneity of the size of the self-selection correction across the 27 studies and point to variability in the mix of organized versus opportunistic screening and in the retention of participants in programs over multiple rounds of screening.

Interestingly, Coldman et al., the authors of the Pan-Canadian Study, indicated that they had conducted a substudy to test for evidence of self-selection bias in their cohort and had found no such evidence (although confidence intervals were wide) [4]. Further, they provided examples of the unreliability of using a self-selection correction that has been imported from a different setting. In such a case it is questionable that a correction relying on data from a very different screening environment should be applied to the published results from this study.

## 3. Conclusions

If a correction for self-selection bias is required it should ideally be constructed utilizing data that are appropriate for the population on which it is to be applied. It is unlikely that the use of a constant derived from the Swedish SOSSEG is appropriate for the data from the Pan-Canadian Study.

## Data Availability

The author would be pleased to share his calculations with other researchers upon reasonable request.
